# It Takes Two to Tango: How a Dysregulation of the Innate Immunity, Coupled With *Candida* Virulence, Triggers VVC Onset

**DOI:** 10.3389/fmicb.2021.692491

**Published:** 2021-06-07

**Authors:** Andrea Ardizzoni, Robert T. Wheeler, Eva Pericolini

**Affiliations:** ^1^Department of Surgical, Medical, Dental and Morphological Sciences with Interest in Transplant, Oncological and Regenerative Medicine, University of Modena and Reggio Emilia, Modena, Italy; ^2^Department of Molecular and Biomedical Sciences, University of Maine, Orono, ME, United States; ^3^Graduate School of Biomedical Sciences and Engineering, University of Maine, Orono, ME, United States; ^4^Graduate School of Microbiology and Virology, University of Modena and Reggio Emilia, Modena, Italy

**Keywords:** vulvovaginal candidiasis (VVC), microbiota, *C. albicans*, neutrophils, secreted aspartyl proteases (Sap), vaginal epithelium, vaginal inflammation, virulence factors

## Abstract

Vulvovaginal candidiasis (VVC) is a symptomatic inflammation of the vagina mainly caused by *C. albicans*. Other species, such as *C. parapsilosis*, *C. glabrata*, *C. tropicalis*, and *C. krusei*, are mainly associated to the recurrent form of the disease (RVVC), although with a lower frequency. In its yeast form, *C. albicans* is tolerated by the vaginal epithelium, but switching to the invasive hyphal form, co-regulated with the expression of genes encoding virulence factors such as secreted aspartyl proteases (Sap) and candidalysin, allows for tissue damage. Vaginal epithelial cells play an important role by impairing *C. albicans* tissue invasion through several mechanisms such as epithelial shedding, secretion of mucin and strong interepithelial cell connections. However, morphotype switching coupled to increasing of the fungal burden can overcome the tolerance threshold and trigger an intense inflammatory response. Pathological inflammation is believed to be facilitated by an altered vaginal microbiome, i.e., *Lactobacillus* dysbiosis. Notwithstanding the damage caused by the fungus itself, the host response to the fungus plays an important role in the onset of VVC, exacerbating fungal-mediated damage. This response can be triggered by host PRR-fungal PAMP interaction and other more complex mechanisms (i.e., Sap-mediated NLRP3 activation and candidalysin), ultimately leading to strong neutrophil recruitment. However, recruited neutrophils appear to be ineffective at reducing fungal burden and invasion; therefore, they seem to contribute more to the symptoms associated with vaginitis than to protection against the disease. Recently, two aspects of the vulvovaginal environment have been found to associate with VVC and induce neutrophil anergy *in vitro*: perinuclear anti-neutrophil cytoplasmic antibodies (pANCA) and heparan sulfate. Interestingly, CAGTA antibodies have also been found with higher frequency in VVC as compared to asymptomatic colonized women. This review highlights and discusses recent advances on understanding the VVC pathogenesis mechanisms as well as the role of host defenses during the disease.

## Epidemiology and Etiology of VVC

*Candida albicans* (*C. albicans*), a member of the endogenous human microbiota, is a dimorphic fungus that dwells in the mucosae of the oropharynx, genital and gastrointestinal tracts of 30–70% of healthy individuals (Odds, 1987; Kauffman, 2006; Pappas, 2006; Pfaller and Diekema, 2007). When the immune system is compromised, *Candida* can behave as an opportunistic pathogen: it causes oral-pharyngeal infections, especially in AIDS patients, but it is also associated with oral cancer (Ramla et al., 2016), diabetes and it can affect terminally ill patients (Venkatasalu et al., 2020). Interestingly, *Candida* is also a leading causative agent of vulvovaginal candidiasis (VVC) in healthy women, even though in this pathology the fungus does not behave as a classical opportunistic pathogen, since VVC is not necessarily linked to an immunodeficient state.

Clinical signs of VVC include itching, burning, pain and redness of the vulva and vaginal mucosa, often accompanied by vaginal discharge. VVC has been reported to affect 70–75% of women of child-bearing age at least once in their lifetime (Blostein et al., 2017; Denning et al., 2018). About 5–8% of the latter can be affected by recurrent VVC (RVVC), mostly due to *C. albicans*. RVVC condition is defined as the occurrence of four or more VVC episodes every year, requiring continual antifungal therapy (Sobel et al., 1998; Cassone, 2015).

By a recent survey, conducted on 284 non-pregnant women over the period February 2016-May 2018 (Yano et al., 2019), 78% of women were reported to have a history of VVC with 34% defined as having RVVC. According to this study, the seriousness of symptoms did not differ among VVC and RVVC women. Interestingly, a high number of participants, irrespective of their history of VVC or RVVC, reported unknown causes for the disease. In the context of vulvovaginal infection, *C. albicans* plays the role of an “immune-reactive commensal” (Jabra-Rizk et al., 2016). Indeed, after the initial attack from *C. albicans*, aggressive neutrophil migration to the vagina is believed to be the main driver of the subsequent acute host inflammatory response. Such a response, in combination with fungal overgrowth within the vaginal environment, ultimately leads to the onset of VVC symptoms (Fidel et al., 2004; Bruno et al., 2015; Pericolini et al., 2015; Jabra-Rizk et al., 2016). Most of the cases of VVC/RVVC are of an endogenous nature because *C. albicans* reaches the vaginal lumen and secretions from the adjacent intestine and anus (Sobel, 2016). Effective anti-*Candida* defense mechanisms, occurring within the vaginal environment, can successfully contain *Candida* as a commensal in an avirulent phase (Sobel, 2007). As mentioned above, VVC and RVVC are not immunodeficiency-associated conditions (Jabra-Rizk et al., 2016), but they are rather linked to predisposing factors such as high oral estrogen administration for contraceptive purposes, hormone replacement therapy, prolonged antibiotic usage, and underlying diabetes mellitus (McClelland et al., 2009; Liu et al., 2013). Nonetheless, *Candida-*specific adaptive immune responses are considered to be protective at the mucosal- and (more specifically) vaginal-*Candida* interface because they keep commensal *Candida* at bay preventing the fungus to switch to a pathogenic condition (LeibundGut-Landmann et al., 2012; Puel et al., 2012; Richardson and Moyes, 2015).

It is important to mention that also non-*albicans Candida* (NAC) species have been identified as etiological agents of 10–30% of VVC cases (Brandolt et al., 2017). Among NAC species, *C. glabrata* is considered as the second leading cause of VVC, followed by *C. parapsilosis, C. krusei*, and *C. tropicalis* (Parazzini et al., 2000; Richter et al., 2005). Back in Kennedy and Sobel (2010) showed that in women with VVC by *C. glabrata*, the fungus was responsible for symptomatology only in 46% of the patients, whereas in the remaining symptomatic women the clearing of the pathogen did not resolve the symptoms caused by inflammation. A similar result was obtained in VVC women with *C. parapsilosis*: in 40% of treated women symptoms persisted despite microorganism eradication. In both cases *C. glabrata* and *C. parapsilosis* were frequently considered “innocent bystanders” (Kennedy and Sobel, 2010). Since neither *C. glabrata* nor *C. parapsilosis* are able to produce true hyphae, these data suggest that other virulence factors must play a key role in the symptomatology. Notably, VVC by NAC species is characterized by milder symptoms, when compared to *C. albicans* VVC (Dan et al., 2002). This was also suggested by Willems and coworkers who showed, by using a murine model of VVC, that whatever the NAC species tested (*C. dubliniensis*, *C. tropicalis*, *C. parapsilosis*, *C. kruzei*, *C. glabrata*, or *C. auris*), both damage and neutrophils recruitment were significantly reduced, when compared to *C. albicans*, despite similar levels of colonization (Willems et al., 2018). However, NAC species can be more resistant to azoles and other antifungal drugs, therefore complicating the VVC treatment (Choukri et al., 2014; Bitew and Abebaw, 2018).

A more recent study revealed the existence of both common and species-specific *Candida* pathogenicity patterns. Specifically, a biphasic host-response by vaginal epithelium has been observed. Such response is characterized by an early activation of mitochondria-induced type I IFN signaling which is shared by all the species considered (*C. albicans*, *C. glabrata*, *C. parapsilosis*, and *C. tropicalis*); the response becomes then species-specific only in the late stages of infection (Pekmezovic et al., 2021). This review will focus on *C. albicans* induced VVC, particularly on the interactions occurring at the fungus-epithelium interface.

## Epithelial Interactions With the Dimorphic Fungus

### Role of the Vaginal Epithelial Barrier

**FIGURE 1:**
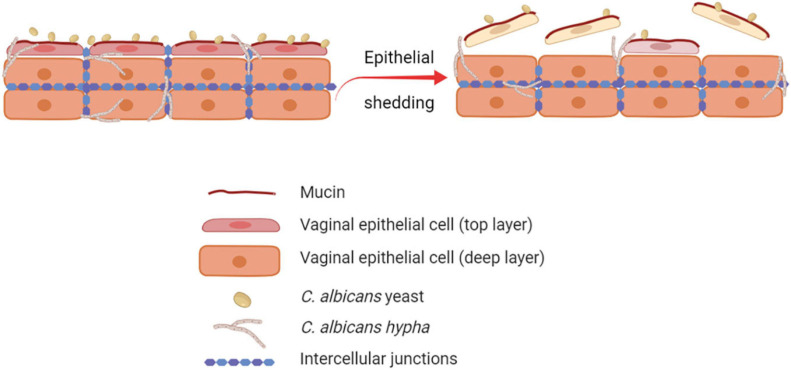
Role of epithelial shedding in restraining *C. albicans* invasion of the vaginal mucosa. The exfoliation of the top layer of epithelial cells into the vaginal lumen (epithelial shedding), restrains vaginal mucosa invasion by *C. albicans*, therefore limiting the fungal burden (created with BioRender.com).

The human vaginal mucosa is lined with many layers of stratified squamous epithelial cells, which are the first host cells to come into direct contact with *C. albicans.* Therefore, they play an important role as the first line of defense by passively and actively restraining *Candida* from invading the underlying tissue. By different mechanisms, the barrier function of the vaginal epithelium prevents adhesion to and invasion of the vaginal mucosa by microorganisms (Naglik et al., 2014; Peters et al., 2014b). Through the epithelial shedding, i.e., the exfoliation of the top layer of epithelial cells into the vaginal lumen, the microorganisms that adhered to or invaded the uppermost epithelial layer are removed, thus limiting the microbial burden (Graf et al., 2019; Figure 1). The precise molecular mechanisms of epithelial shedding in the context of VVC are yet to be unraveled. The direct contact of *C. albicans* with the epithelial cell surface is also hindered by mucin, a molecule responsible of the mucus layer coating the epithelial cells (Patton et al., 2000; Cassone and Cauda, 2012; Cassone, 2015). In addition, epithelial integrity is ensured by strong interepithelial cell connections, which prevent mucosal invasion by *C. albicans* (Yan et al., 2013).

### Role of Fungal Virulence Factors in Overcoming the Epithelial Tolerance Threshold

**FIGURE 2:**
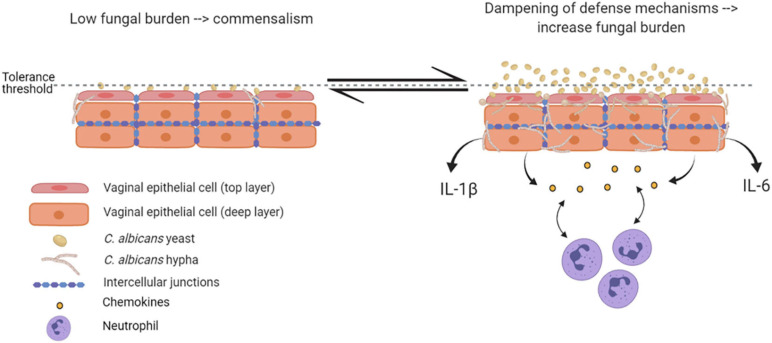
Epithelial tolerance in murine model of VVC. *C. albicans* in its yeast form is tolerated by epithelial cells, which do not trigger any immune response. However, upon dampening of local defense mechanisms, fungal burden increases, yeasts-to-hyphae transition occurs, and the epithelial tolerance threshold is exceeded; as a consequence, epithelial cells trigger an intense inflammatory response, associated with proinflammatory cytokines and chemokines release and neutrophils recruitment (created with BioRender.com).

Epithelial immune responses *in vitro* depend on both fungal morphotype and load. Yeast cells represent the phenotypic form responsible for the colonization of the vagina. Germinated yeasts, which produce hyphae, can be found most commonly in symptomatic vaginitis (Sobel et al., 1998; Pericolini et al., 2018; Roselletti et al., 2019b). As shown in Figure 2, in healthy subjects, *C. albicans* occurs in low numbers as a commensal on the mucosal surfaces. Based on *in vitro* experiments and murine models, evidence exists that *C. albicans* is tolerated by epithelial cells without triggering an epithelial immune response (Figure 2). Indeed, the tolerance threshold prevents the activation of a potentially damaging response by proinflammatory cytokines. When local defense mechanisms are dampened, both *C. albicans* burden and virulence increase. In this scenario, the increased fungal burden may exceed the tolerance threshold of epithelial cells; as a consequence, the latter may become susceptible to the fungus and trigger an intense inflammatory response, leading to pro-inflammatory cytokines (such as IL-1β and IL-6) production and release (Cassone and Cauda, 2012; Naglik et al., 2014). Contrarily, in women experimentally challenged with *C. albicans*, such correlation has not been observed (Fidel et al., 2004), suggesting the existence of alternative mechanisms that allow for circumventing the breaking of epithelial tolerance. Colonization of the vagina requires adhesion to vaginal epithelial cells (King et al., 1980). The initial adhesion likely occurs between yeasts and epithelial cells and this interaction stimulates the formation of hyphae that are generally considered to be the most adherent morphology of *C. albicans* (Naglik et al., 2011). Formation of hyphae is associated with the expression of characteristic hyphae-associated genes encoding virulence factors. These include hyphal wall protein 1 (Hwp1), Agglutinin-like sequence 3 (Als3), secreted aspartyl proteases 4, 5 and 6 (Sap4, 5, and 6), the hyphae-associated proteins Extent of Cell Elongation Protein 1 (Ece1) and Hyphal Regulated Cell Wall Protein 1 (Hyr1) (Sudbery, 2011). Once hyphae are formed, adhesins expressed exclusively on the hyphal cell surface further strengthen the adhesion processes (Moyes et al., 2015). The adherent hyphae can also promote invasion of the vaginal epithelium.

*Candida* has evolved to be retained as a commensal on the healthy surface of host tissues (Gow and Hube, 2012). It is assumed that during both commensalism and pathogenesis there is a certain degree of invasion into host cells, which is required to maintain a foothold on the cell surface and to avoid being sloughed off from epithelial surfaces (Gow and Hube, 2012). It has been suggested that these unsuccessful invasion attempts lead, over evolutionary time, to the selection of traits that prepare invading cells for infection, termed “predictive adaptation” (Brunke and Hube, 2014). These adaptations likely include regulation of yeast to hyphal switching and expression of hyphal-associated virulence factors (Whittington et al., 2014).

### Mechanisms of Vaginal Epithelial Invasion by *C. albicans*

**FIGURE 3:**
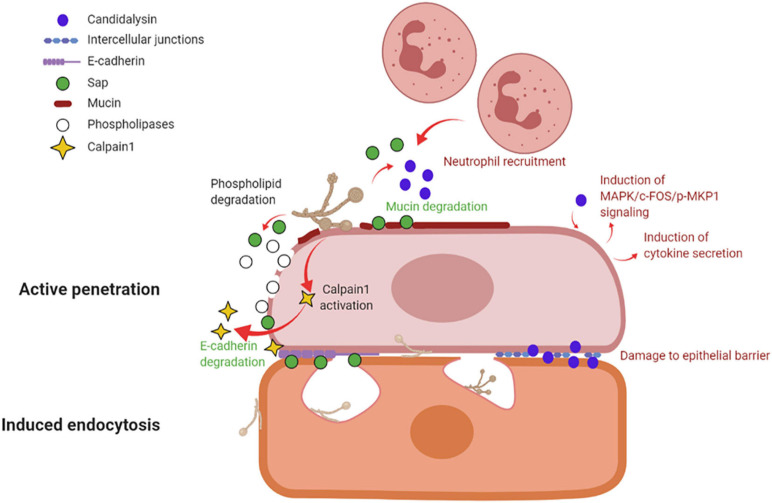
Mechanisms of vaginal epithelial invasion by *C. albicans*: active penetration and induced endocytosis. By active penetration, *C. albicans* actively invades the uppermost layers of epithelial cells from where the fungus passes through the intercellular junctions and invades the deeper epithelial layers through either active penetration or induced endocytosis. *C. albicans* anchors to epithelium via its Als3 adhesin; in addition, the secretion of molecules such as Sap, phospholipases and candidalysin, play a part in fungal invasion. Sap contributes to mucin and intercellular junctions’ degradation, which facilitates fungal invasion, and similarly to candidalysin is responsible for neutrophils recruitment. Candidalysin induces MAPK/c-FOS/pMKP1 signaling and triggers proinflammatory cytokines secretion. The activation of calpain-1 further facilitates *C. albicans* invasion of deep epithelial layers. The second mechanism of epithelial invasion, induced endocytosis, is mediated by epithelial cells. It begins with the adhesion of *C. albicans’* Als3 to the epithelial surface and it is followed by pseudopod formation and fungal uptake inside the cell (created with BioRender.com).

By *in vitro* studies it has been demonstrated that *C. albicans* invades host cells by means of two distinct mechanisms: induced endocytosis and active penetration (Wächtler et al., 2012; Figure 3).

Induced endocytosis is mediated by epithelial cells. The interaction between fungal invasion proteins, expressed on hyphal surface, and receptors on epithelial cells is followed by pseudopod formation and fungal uptake. It is a passive process, and both killed and viable hyphae are endocytosed, although the endocytosis of killed hyphae is not as effective as for viable cells. Differently, active penetration requires fungal viability and allows *C. albicans* to invade cells or to pass through the intercellular junctions between epithelial cells (Naglik et al., 2011). In the vaginal lumen, upholstered by stratified squamous epithelium, the uppermost epithelial layers are thought to be non-proliferative and functionally inactive (Naglik et al., 2011; Höfs et al., 2016). Therefore, it is likely that the initial mucosal invasion by *C. albicans* is mainly mediated by a process of active penetration, which allows the fungus to reach deeper epithelial layers (Naglik et al., 2011). This mechanism also includes the release of the fungal toxin candidalysin (Ho et al., 2021). Here, viable and active epithelial cells allow for the invasion by a mechanism of induced endocytosis. Thus, both processes are likely to play complementary roles, *in vivo*, during the invasion of vaginal mucosal tissues (Naglik et al., 2011; Höfs et al., 2016). Moreover, evidence exists of vaginal epithelial invasion of *C. albicans* in a mouse model of VVC (Bruno et al., 2015).

Several molecules, like the adhesin Als3 and *C. albicans* secreted enzymes such as Sap or phospholipases contribute to *C. albicans* invasion. Als3 plays a role during invasion by anchoring *C. albicans* to the host cell; differently, Sap mediate fungal active penetration by facilitating inter-epithelial invasion. The latter occurs via degradation of E-cadherin, an important component of intercellular junctions, and host proteins like mucins, resulting in the loss of epithelial integrity and barrier function (Colina et al., 1996; Frank and Hostetter, 2007). Moreover, *C. albicans* can invade the deep layer of epithelium by activating epithelial calpain1, a Ca^2+^-dependent cysteine protease involved in many cellular processes. Activated calpain1 cleaves E-cadherin promoting *C. albicans* tissue invasion (Xu et al., 2016). Phospholipases degrade phospholipids of the cell membrane (Ghannoum, 2000). Moreover, candidalysin has been shown to cause damage, to induce MAPK/c-Fos/p-MKP1 signaling activation, cytokine secretion and to drive immunopathology via the neutrophils’ strong recruitment in the vaginal environment (see below and Figure 3).

Notably, the vagina harbors many species of microorganisms that interact with both fungal cells and epithelial cells and therefore may play a role (as yet unraveled) in the VVC onset.

## Role of the Microbiota in VVC Onset

### Composition of Vaginal Microbiota

Although VVC is mainly a mono-microbial infection, regulated by the above-mentioned interactions between *C. albicans* and the epithelium, the disease is multifactorial. Disruption of the vaginal microbiota may facilitate increased virulence and high burden of *Candida* (Sobel, 2007). In parallel, the vigorous local inflammatory response associated with co-infection may orchestrate the development of symptoms. *Candida* vaginitis occurs in the context of a complex commensal microbial environment. The vulvovaginal area harbors a diverse and variable microbiota, including bacteria and fungi, but some generalizations can be made on the composition of a healthy vaginal microbiota. Interestingly, ethnicity is one of the features contributing to influence the composition of the vaginal microbiota (de Vos et al., 2012; Smith and Ravel, 2017). Molecular studies, carried out on 16S rRNA, have demonstrated that variations occur in the relative proportion of specific vaginal bacteria, even among healthy, asymptomatic women (Zhou et al., 2004; Ravel et al., 2011). Those studies demonstrated that the vaginal lumen has a complex and dynamic resident microbiota, of which *Lactobacilli* are the most represented genus.

Notably, the role of vaginal microbiota in VVC is still largely unexplored and the literature reports are often contradictory. On one hand, some microbiota patterns suggest an altered vaginal microbiome in VVC patients (Hillier et al., 1992). In particular, *Lactobacilli* have been reported to be essential for vaginal health. Vaginal microbial communities where they predominate have been suggested to indicate a healthy microbiome, whereas VVC has been associated to disruption of such microbiota composition (Cassone, 2015). These data appear in contrast with others, reporting the lack of significant differences in the *Lactobacillus* spp. colonization of women with or without VVC (Sobel and Chaim, 1996; Pericolini et al., 2018). Since more powerful next-generation sequencing (NGS) techniques have been applied to this matter, the only clear result has been the demonstration of a high variability in the VVC vaginal microbiome among patients (Liu et al., 2013).

The most represented species of *Lactobacilli* occurring in the vaginal environment are *L. iners*, *L. crispatus*, *L. gasseri*, and *L. jensenii* (Ravel et al., 2011). *L. iners* has been found to be ubiquitous even during dysbiosis, while *L. crispatus* has been mainly associated to a healthy microbiota. *L. gasseri* or *L. jensenii* have been found to occur less frequently (van de Wijgert et al., 2014). Many studies have reported *Lactobacillus* to be the dominant genus not only in healthy women but also in women with VVC, whereas it is depleted in bacterial vaginosis (Macklaim et al., 2015). While the association of a healthy status with the presence of *Lactobacilli* remains unexplained, several potential mechanisms could instead account for the inhibition of VVC by bacterial colonization.

### Mechanisms of VVC Inhibition by *Lactobacilli*

**FIGURE 4:**
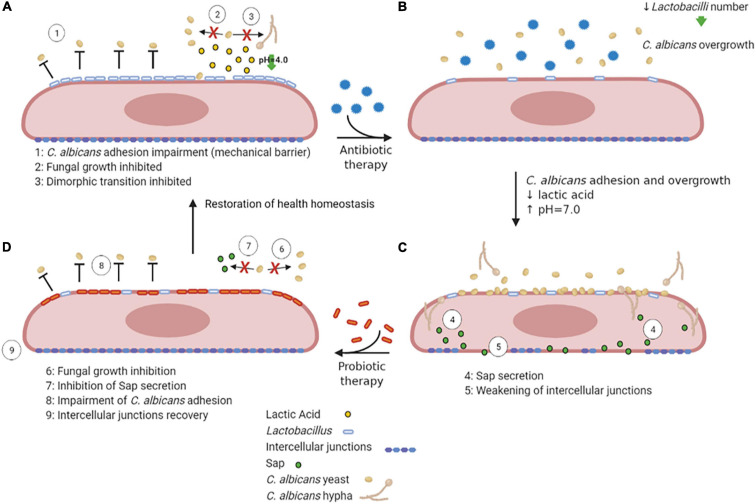
VVC inhibition by *Lactobacilli*, antibiotic therapy dysbiosis and probiotic therapy recovery. A healthy vaginal epithelium is colonized by *Lactobacilli*; the latter outcompete *C. albicans* for adhesion through several mechanisms, such as production of lactic acid, which also inhibits *C. albicans* yeast-to-hypha transition **(A)**. Upon antibiotic use, the decrease in *Lactobacilli* number is followed by an increase in intravaginal pH, followed by *C. albicans* overgrowth **(B)**. The dismicrobism allows for *C. albicans* overgrowth and adhesion; moreover, the Sap secretion by the fungus causes the weakening of epithelial intercellular junctions **(C)**. The administration of a probiotic therapy restores *Lactobacilli* colonization, impairs *C. albicans* adhesion and inhibits Sap secretion by the fungus, allowing the recovery of intercellular junctions’ integrity, ultimately allowing to restore the health homeostasis status **(D)** (created with BioRender.com).

*Lactobacilli* may inhibit VVC onset by a number of mechanisms, including the production of lactic acid, hydrogen peroxide and bacteriocins (de Vos et al., 2012). They bind tightly to vaginal epithelial cells without giving rise to pathology; therefore, they may outcompete *C. albicans* simply for adhesion to the host cells. In addition, they are capable of thriving in a low-oxygen environment such as the one occurring in the vagina. Moreover, the production of lactic acid by these bacteria causes acidification of the vaginal environment (Green et al., 2015). Not only *Lactobacilli* are acidogenous, they are also acidophilic, since they easily tolerate the acidic pH of the vagina that they contribute to create. Vaginal pH is near 4.0 for most of the menstrual cycle, and it briefly rises to almost 7.0 only when menstruation begins. The acidic pH inhibits *C. albicans* hyphal growth and may play an important role in limiting the invasion as well as the expression of hyphal-associated virulence factors. This hypothesis is supported by the observation that several *Lactobacilli* prevent *C. albicans* hyphal growth and exert a direct antifungal activity (Noverr and Huffnagle, 2004; Strus et al., 2005; Huang, 2012; Liang et al., 2016; Zangl et al., 2020; Figure 4A). It follows that resident microbiota naturally promotes a balanced and protective immunity against VVC. *C. albicans*, competes with both bacteria and epithelial cells for nutrient acquisition and this forces the fungus to be metabolically flexible (Whittington et al., 2014).

### Probiotic Therapy Against VVC

Further support for the idea that commensal microbes can tip the balance in VVC comes from studies that have linked antibiotic use to VVC and conversely linked probiotic treatments to the prevention or treatment of the disease. Indeed, during or after antibiotic therapy, when *Lactobacillus* numbers are reduced and the pH increases to values close to neutrality (Peters et al., 2014b), such infections frequently arise. Antibiotics perturb the bacterial community and cause a fungal overgrowth (Figures 4B,C). Unsurprisingly, VVC commonly follows prolonged antibiotic treatment, indicating that the vaginal microbiota normally prevents heavy colonization and invasive disease (Liu et al., 2013). While in some studies the association between antibiotic treatment and occurrence of VVC was not observed (Barbone et al., 1990; Geiger and Foxman, 1996), other studies report a high VVC prevalence in women that had undergone antibiotic therapies (Spinillo et al., 1999). Yano and coworkers identified antibiotic use as the highest risk factors for VVC even though for a high number of women included in the study the etiopathogenesis of VVC or RVVC was idiopathic (Yano et al., 2019).

Probiotic therapy, where *Lactobacilli* can be orally administered (Russo et al., 2019) or directly applied to the vagina (Pendharkar et al., 2015), when associated to conventional antimicrobial and/or antifungal therapies, has been successful in helping patients with vaginitis or vaginosis with an excellent overall safety record. Moreover, it has been demonstrated that in mice with vaginal infection by *C. albicans*, intravaginal treatment with *Saccharomyces*, used as a probiotic, provides beneficial therapeutic effects mediated by a synergism between mechanical (i.e., formation of a barrier on epithelium and induction of *Candida* coaggregation) and biological effects (i.e., active inhibition of *Candida* virulence factors such as Sap secretion) (Pericolini et al., 2017; Wilson, 2017; Figure 4D). *Lactobacilli* may also enhance the natural barrier function of epithelial cells by the induction of an increased production of structural and transmembrane proteins in the vicinity of the tight junctions, which can be weakened due to inflammation or pathogen virulence factors (Saulnier et al., 2008). Weakening of this barrier induces macrophage recruitment and may enhance the sloughing off of epithelial cells, leading to vaginal discharge. Given that these probiotics can directly inhibit the growth of pathogens such as *C. albicans* and can modulate human immune-responses, they appear to be a promising novel option for antifungal therapy (de Vos et al., 2012).

### Coassociation Between *C. albicans* and Possible Pathogenic Microorganisms

The interactions between *C. albicans* and other vaginal microbes can affect VVC by different mechanisms, i.e., direct competition, antagonism or synergy, or through the regulation of immune responses that may lead to tolerogenicity or inflammation.

Both *Gardnerella vaginalis* and *C. albicans* commonly occur in healthy women, where they can cause opportunistic vaginitis, vaginosis and/or vaginal candidiasis. Overall, “normal” vaginal health may require dense vaginal populations of *Lactobacillus* that reduce colonization by *G. vaginalis* and *C. albicans* (Wilson, 2005). In addition to blocking adhesion, lactic acid from the bacteria create acidic conditions (pH 4) that inhibit filamentous growth. Thus, resident vaginal bacteria inhibit fungal growth, produce fatty acids, and compete for epithelial cell binding sites and for nutrients. Recent studies demonstrated an important role of Group B *Streptococcus* (GBS), providing evidence of reciprocal, synergistic interplay between GBS and *C. albicans* (Pidwill et al., 2018). Moreover, experimental co-inoculation of GBS with *C. albicans* can promote bacterial adhesion to the bladder epithelium in a *C. albicans* adhesin-dependent manner (Shing et al., 2020).

It has been shown that *C. albicans* is capable to form biofilms on the vaginal mucosa *in vivo* and *ex vivo* (Harriott et al., 2010). This raises interesting questions about the precise role played by biofilms, i.e., whether the presence of a biofilm regulates *C. albicans* pathogenesis or if it rather influences the host response. It is still unknown if biofilms affect colonization of bacterial species in vagina contributing to bacterial pathogenesis or drug resistance. In nature, biofilms are most likely polymicrobial and therefore they represent a complex signaling environment for interspecies and interkingdom selective pressure. For this reason, it is important to understand whether the presence/occurrence of two or more species such as *G. vaginalis* and *C. albicans* predisposes the host to colonization or infection (Jabra-Rizk et al., 2016). Evidence exists that *C. albicans* can form both mono- and polymicrobial biofilms that may exacerbate VVC and make treatment more difficult (Harriott and Noverr, 2011). One potentially pathogenic co-colonizer of biofilms is *G. vaginalis*, which causes vaginosis and forms biofilms on the vaginal mucosa (van der Meijden et al., 1988; Scott et al., 1989; Swidsinski et al., 2008). However, it is still unknown if *C. albicans* and *G. vaginalis* can be part of the same vaginal polymicrobial biofilms in VVC and RVVC, playing thus an immune-pathogenic role. There is also the risk of mono- or poly-microbial biofilm formation on abiotic surfaces of vaginal or intrauterine devices, which would contribute to incidence and recurrence of genital infections (Harriott and Noverr, 2011).

The remaining part of the review will focus on the role of immune response in the pathogenesis of VVC.

## Host-Fungal Interaction Induces Non-Protective Immune Response

### PRRs Recognition of *C. albicans* PAMPs

*C. albicans* interacts with the host as a commensal or as a pathogen, depending on the microbial environment, the host immune response and fungal virulence. Thus, both microbial and host factors are responsible for inducing tissue damage, including the host immune response that appears to worsen fungal-mediated damage (Casadevall and Pirofski, 1999, 2003). Although fungal virulence factors are important for the initial onset of VVC, the propagation of disease is largely mediated by the host immune system. Immune detection of *C. albicans* occurs through pattern recognition receptors (PRRs) of innate immune cells. PRRs such as Toll-like (TLR) and C-type lectin receptors (Dectin-1 and -2), interact with pathogen associated molecular patterns (PAMPs) on *C. albicans* cell wall, such as glucans and mannans, leading to the secretion of inflammatory cytokines (Jabra-Rizk et al., 2016). Even though the mechanisms of *C. albicans* recognition have been described, the event(s) turning the fungus an enemy to women immune system are still obscure; whatever the mechanism(s) responsible for such “enmity,” the result is the onset of a powerful inflammatory response at the mucosal level.

By analyzing healthy women, asymptomatic *C. albicans* carriers, and symptomatic patients with VVC, Roselletti and co-workers showed that inflammatory signals are activated in epithelial cells only from symptomatic and asymptomatic pseudohyphae/hyphae carriers but not from the asymptomatic yeast carriers. Their conclusion is that the presence of pseudohyphae/hyphae is a prerequisite to determine VVC, but it may be not sufficient to induce the pathologic process (Roselletti et al., 2019b). Therefore, the formation of hyphae seems to be not sufficient *per se* to trigger this response (Pericolini et al., 2018; Roselletti et al., 2019b). If it is not clear the role of hyphae *per se* to induce the pathology, there are numerous evidences from different research groups that clarify the role of neutrophils as the main responsible of the VVC symptoms. It is likely that a communication system may exist between immune cells, such as neutrophils, and fungal cells within the vaginal environment.

**FIGURE 5:**
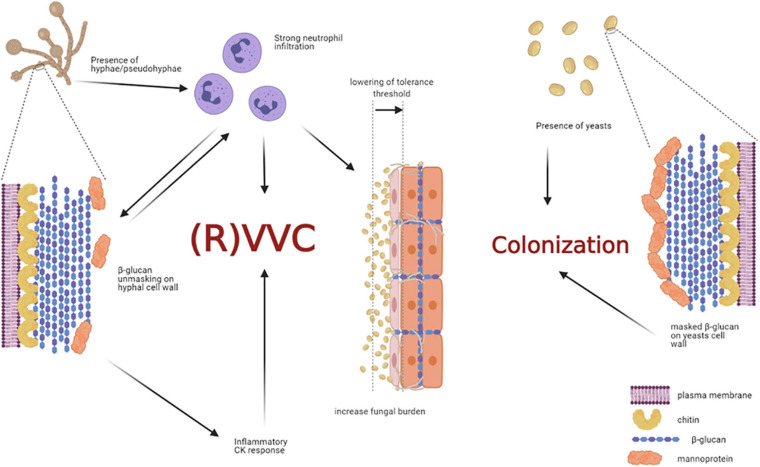
Non-protective immune response triggered by host-fungal interaction. The presence of *C. albicans* hyphae is a prerequisite for VVC onset, but it may not be sufficient to induce the pathologic process. Indeed, hyphae cause neutrophils recruitment; the latter drive fungal cell-wall changes, such as β-glucan unmasking, which in turn elicits an inflammatory cytokine response. In addition, the interaction of *C. albicans* hyphae with vaginal epithelial cells further contributes to neutrophils’ recruitment, by overcoming the epithelial tolerance threshold. The neutrophils, which trigger an intense inflammatory response, are unable to clear the fungus and they significantly contribute to VVC onset. Indeed, such massive neutrophils migration to the vagina and the subsequent acute host inflammatory responses are retained to be the main responsible for the symptoms of VVC. Differently, the presence of *C. albicans* yeasts do not cause neutrophils recruitment, β-glucan unmasking does not occur, and the host-fungal interaction ends up in a colonization process (created with BioRender.com).

In our recent paper we have shown that women with symptomatic VVC have much higher percentages of hyphal fragments and high neutrophil infiltration in their vagina, as compared to asymptomatic colonized women (Pericolini et al., 2018). Moreover, we found that usually β-glucan (a fungal PAMP with proinflammatory activity) is largely masked from immune-recognition, especially on yeasts. Differently, β-glucan has been found to be less masked (and thus more available) only in symptomatic patients with strong neutrophil infiltration, implicating neutrophils as the possible drivers of such fungal cell wall changes (Pericolini et al., 2018). Indeed, it has been demonstrated in *ex-vivo* experiments that when neutrophils attack *C. albicans*, hyphal β-glucan (normally masked in the cell wall from host recognition) is unmasked (Hopke et al., 2016). In addition, such remodeling process of cell wall architecture further enhances recognition and elicits an inflammatory cytokine response much greater than yeast β-glucan (Figure 5). However, the role of β-glucan unmasking in driving the inflammation during *C. albicans* vaginitis remains unresolved because it is unknown if this type of neutrophil-mediated cell wall remodeling enhances pro-inflammatory immune responses during vaginitis. It follows that even these data do not help understand why the PRR-PAMP interactions at vaginal level train the immune response toward the disease or the asymptomatic status. Finally, as it will be better explained in the next chapter *Candida* PAMPs (i.e., β-glucan) can be modulated by changes in carbon source (Ballou et al., 2016).

### Role of Neutrophils in VVC Onset

In symptomatic VVC, a massive neutrophil migration to the vagina and the subsequent acute host inflammatory responses are presently maintained to be the main responsible causes for mucosal damage. Murine studies show that the neutrophil response is triggered by the interaction of *C. albicans* hyphae with vaginal epithelial cells (Peters et al., 2014a); yet, these recruited cells have no apparent ability to clear *Candida* (Sobel et al., 1998; Fidel et al., 2004). Moreover, in mice infected with hyphae-deficient transcriptional regulator mutant strains the inflammatory signals, including neutrophils recruitment, is significantly reduced as compared to mice infected with hyphae-competent strains; however, the fungal burden does not differ in the two conditions. Interestingly, depleting neutrophils with neutralizing antibodies did not reduce fungal load, nor vaginal mucosal damage during infection (Peters et al., 2014a). All these findings suggest that neutrophils may contribute more to the symptoms associated with vaginitis (i.e., damage) than they contribute to protection against the disease (Fidel, 2004; Yano et al., 2010; Peters et al., 2014b; Figure 5). This concept is especially hard to understand given the well-characterized ability of neutrophils to kill *C. albicans* yeast and hyphae *in vitro* through phagocytosis and NETs (Rubin-Bejerano et al., 2003; Urban et al., 2006). Clinical studies by Fidel’s research group, conducted in volunteer women without a previous VVC episode or with infrequent VVC episodes and experimentally challenged with live *C. albicans*, showed that symptomatic infection correlates with both vaginal infiltration of neutrophils and high vaginal fungal burden (Fidel et al., 2004). In contrast, in women asymptomatically colonized, the absence of neutrophilic inflammatory response was coupled with a significantly lower fungal burden. In accordance with such data, vaginal lavage fluid from women with a symptomatic infection, but not from those asymptomatically colonized, has been shown to promote neutrophils’ chemotaxis (Fidel et al., 2004). Once again, these results confirm the idea that symptomatic infection is mostly mediated by host factors, rather than by a direct fungal damage.

Epithelial cells are another element on the host side that can play a role in the onset of VVC. Indeed, vaginal epithelial cells have a tolerance threshold for *Candida* that varies among women and influences the clinical outcome (Fidel, 2007). As mentioned above, in the presence of predisposing factors, when the number of *C. albicans* cells increases particularly in the hyphal form, this threshold can be exceeded, and epithelial cells will trigger an intense inflammatory response. Consistent with this hypothesis, Fidel’s research group has deduced that in women with RVVC the epithelial cells are more sensitive to *Candida*, as compared to epithelial cells of women with infrequent history of VVC. The reason behind such higher epithelial sensitivity is yet unknown. In women without history of VVC, the vaginal epithelial cells are insensitive to large numbers of *Candida*, the threshold is seldom reached, and the inflammatory response rarely occurs; consequently, these women remain asymptomatic (Yano et al., 2012).

### *In vivo* Models to Study VVC

While human challenge experiments remain an important means to study VVC, it must be considered that experimental human vaginal infection does not accurately mirror the natural process of infection acquisition. In the human challenge model, symptomatic infection is independent on the stage of the menstrual cycle (Fidel, 2004), but in natural infections VVC tends to occur or worsen in the presence of higher levels of estrogens and progesterone, the concentration of which increases in the luteal phase of the cycle (Cassone and Sobel, 2016). To verify the mechanistic basis for the pathogenesis of VVC, work has relied on the well-established estrogen-dependent rat (Cassone et al., 1999) and mouse models (Pietrella et al., 2011; Yano and Fidel, 2011; Pericolini et al., 2015). Longitudinal monitoring of VVC infection in mice was made possible by using a strain of *C. albicans* expressing luciferase. This protocol allowed for the non-invasive imaging of VVC burden in order to monitor the extent and duration of the infection in vagina (Vecchiarelli et al., 2015). Both rodent models show some parallels with the immunological and physiological properties of human VVC (De Bernardis et al., 1997; Fidel et al., 1999), although the main differences consist in a vaginal pH close to neutrality (in mice) and the fact that *C. albicans* is not a normal member of the vaginal microbiota of rodents (mice and rats) (Vecchiarelli et al., 2015). Furthermore, and in contrast to rodent models, no keratinization of vaginal epithelial surface has ever been reported in women (Sobel, 2016). Although there are limits to both the human intravaginal challenge model and the rodent vaginitis models, it is hoped that a combination of these approaches will elucidate the relationship between host response and symptomatic infection.

### Genetic Studies on VVC Pathogenesis

Most cases of VVC occur in women without any known risk factors, suggesting that also a genetic predisposition may occur in individual patients, in combination with a strong environmental influence (Cassone, 2015; Yano et al., 2019). Such genetic predisposition, hypothesized to be due to the contributions of several susceptibility genes, may play a key role in the VVC onset. Literature data suggest that a homozygous polymorphism (Tyr238X stop codon) in Dectin-1 (the main β-glucan receptor) may confer susceptibility to RVVC (Ferwerda et al., 2009). However, in a murine model of VVC, where Dectin-1 is not expressed on vaginal epithelial cells (Dectin-1^–/–^ mice), no changes in neutrophil recruitment or fungal burden have been described (Yano et al., 2014). More recently, the SIGLEC15 gene polymorphism has been associated with RVVC. This study performed in a cohort of RVVC and healthy women has revealed an altered cytokine profile after PBMC stimulation with *C. albicans* (Jaeger et al., 2019). Other polymorphisms predisposing to RVVC have been reported, implicating mannose-binding lectin, IL-4 and CARD9 in resistance to VVC (Babula et al., 2003, 2005; Liu et al., 2006; Smeekens et al., 2013). These genetic studies also provide independent validation for the importance of the NLRP3 inflammasome in RVVC development and progression (Jaeger et al., 2016).

Early microarray studies analyzed *C. albicans* gene expression during invasion of epithelial cells (Moyes et al., 2010; Ikuta et al., 2012), as well as epithelial cell responses to infection by *C. albicans* (Sandovsky-Losica et al., 2006; Zakikhany et al., 2007; Park et al., 2009). More recently, the more sensitive technique of RNA-seq has been employed to characterize the transcriptomes of both *C. albicans* and host cells during VVC infection in human subjects. By this technique, a significant transcriptomic evidence for the involvement of platelet-derived growth factor BB (PDGF BB), a serum protein that stimulates cellular migration, was found (Liu et al., 2015). Even though PDGF BB has been found to be activated during clinical episodes of VVC (Demoulin and Essaghir, 2014), it is still unknown how its signaling acts during human VVC. It has been hypothesized that it may enhance fungal invasion through endocytosis (Liu et al., 2015). RNA-seq analysis was also applied by Bruno and coworkers to mouse vaginal *C. albicans* infection. Remarkably, this study confirmed and extended previous work where involvement of the NLRP3-inflammasome in driving immune-mediated damage in VVC had been already described (Bruno et al., 2015).

## Leading Actors of Symptomatic Inflammation: Secreted Aspartyl Proteases (Sap), NLRP3-Inflammasome Activation, Candidalysin and β-Glucan Unmasking

Since human challenge studies imply that inflammation, rather than fungal burden, drives the symptomatic disease, recent studies have focused on host and pathogen factors, both implicated in inducing inflammation *in vitro* and *in vivo*. By these works, Sap, inflammasome activation, candidalysin and β-glucan unmasking have been identified as key fungal-derived inflammatory triggers. Host inflammasome activation, in particular, has been acknowledged as a key host pathway driving immune recruitment, activation and inflammation (Pericolini et al., 2015; Moyes et al., 2016). Transcriptomic work in human infection has further confirmed the importance of inflammasome activation and specific signaling pathways in human VVC (Liu et al., 2015).

### Secreted Aspartyl Proteases (Sap)

Two different investigations by Roselletti and coworkers carried out on human samples, showed that NLRP3 inflammasome activation in epithelial cells from VVC women is coupled with upregulations of key genes involved in *C. albicans* virulence such as: yeast-associated gene *SAP2* and the hyphae associated genes *SAP5*, *SAP6*, *HWP1*, and *ECE1* (Roselletti et al., 2017, 2019a). Such studies have allowed to expand previous results obtained from murine studies. In particular, work from two independent groups, which used mouse models of VVC, showed that Sap play an important role in vaginal inflammation. Pericolini and coworkers demonstrated that a significant proportion of the early (24 h) vaginal inflammation was found to be mediated by Sap2 and/or the closely related, antigenically cross-reactive Sap1 and Sap3, typically associated with yeast cells (Pericolini et al., 2015). In addition, data from Bruno and coworkers implicated *SAP5* (typically hypha-associated) in the late immune-pathogenic activity of *C. albicans* (Bruno et al., 2015). Both models point to the idea that several Sap play an important role in the induction of two classical signs of inflammation during VVC, i.e., neutrophil influx and pro-inflammatory cytokine expression, particularly IL-1β (Bruno et al., 2015; Pericolini et al., 2015). In line with this idea, by using the CD1 outbred mouse model, early *C. albicans*-induced inflammation in vaginal environment was dampened by treatment with either Anakinra, a selective antagonist of IL-1 receptor, or with an anti-Sap2 neutralizing antibody. Notably, inhibition of Sap2-induced vaginal inflammation did not affect the level of vaginal colonization in its early stages (Pericolini et al., 2015). These results correlate well with the data by Bruno VM et al., who showed that by using a *C. albicans* strain lacking *SAP5* both neutrophil recruitment and IL-1β secretion were significantly reduced, whereas colonization level was not affected (Bruno et al., 2015). Therefore, during experimental VVC the Sap-induced impairment of neutrophil recruitment and cytokine secretion has been demonstrated to be sufficient to block the inflammation, without affecting the fungal load (Bruno et al., 2015; Pericolini et al., 2015). The observation that neutrophil recruitment can be decoupled from the level of vaginal fungal colonization confirms the idea that neutrophils, instead of playing a predominantly protective role during *Candida* vaginal infection, rather exacerbate the disease and contribute to the symptoms (Naglik et al., 2014). Therefore, following an initial insult from *C. albicans*, the propagation of disease is largely mediated by the overreaction of the host immune system rather than by the fungus itself (Jabra-Rizk et al., 2016).

These findings provide some rationale for development of a Sap2-based virosomal vaccine for VVC (PEV7). The vaccine completed a phase 1 clinical trial, is in further stages of immunogenicity and toxicity testing, and may be used in a combined anti-*Candida* vaccine (Pevion, NovaDigm) (Cassone, 2013; De Bernardis et al., 2018). Furthermore, this raises the question of whether the expression of *SAP2* could be considered as an early biomarker of *C. albicans* transition from commensal to pathogenic lifestyle. However, future studies should be devoted to clarifying this issue in by longitudinal studies of human vaginal samples spanning the time prior to symptom onset as well as the time of symptomatic disease and the resolution phase.

**FIGURE 6:**
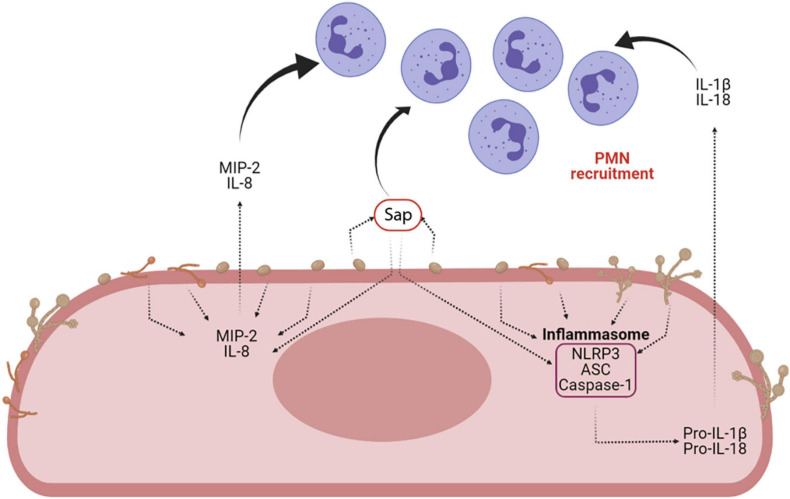
Sap-induced inflammasome activation and inflammasome-dependent cytokine production. Sap secretion by *C. albicans* is one of the main virulence factors of the fungus. Sap can recruit neutrophils both directly and indirectly, through the activation of the NLRP3 inflammasome and the induction of proinflammatory cytokines (i.e., IL-1β and IL-18) and chemokines (MIP-2 and IL-8) (Created with BioRender.com).

Sap play a key role in the induction of cytokine secretion by different immune cells. They drive lysosomal rupture-dependent IL-1β release (Pietrella et al., 2013; Pericolini et al., 2015; Tavares et al., 2015), induce type I IFN production and activate caspase-11, which in turn cooperates with caspase-1 to maximize IL-1β maturation (Gabrielli et al., 2015; Figure 6).

IL-1β is an inflammatory cytokine that, together with IL-18 and IL-22, is critical for both innate defense and adaptive Th17/Th1/Treg responses. The link between IL-1β and adaptive immune response activation in VVC has been shown by studies conducted in murine models where the IL-18 and IL-22 deficiency was associated to an increased susceptibility to VVC (van de Veerdonk et al., 2011; Borghi et al., 2019).

The role of Th17 responses in VVC is currently controversial.

Indeed, a relevant role for IL-17 in VVC has been reported, suggesting a protective role played by this cytokine in the vaginal compartment (Pietrella et al., 2011). Moreover, the presence of IL-17 correlates with increased production of antimicrobial peptides by epithelial cells (Pietrella et al., 2011). However, beyond these data, the role of the IL-17 pathway during VVC remains unclear, with conflicting reports from human data and mouse models. A recent paper by Peters and coworkers has shown that mice lacking IL-17RA, Act1, or IL-22 have no altered VVC susceptibility or immunopathology, regardless of estrogen administration. Hence, these data support the emerging consensus that Th17/IL-17 signaling axis plays a non-essential role in the immunopathogenesis of VVC (Peters et al., 2020).

### Sap-Induced Inflammasome Activation

Recent work has helped explain how Sap provoke inflammatory neutrophil recruitment and cytokine induction, suggesting that Sap trigger neutrophilic chemokine production by the host and induce NLRP3-inflammasome activation (Pericolini et al., 2015; Gabrielli et al., 2016). A combination of *in vitro*, *ex vivo* and *in vivo* works demonstrated that Sap recruit neutrophils, both directly and indirectly. They directly induce neutrophil migration, independently from their proteolytic activity, and their enzyme activity indirectly induces, by favor Sap entering the epithelial cells, the production of chemo-attractive cytokines such as macrophage inflammatory protein 2 (MIP-2) and IL-8 from vaginal epithelial cells to further enhance neutrophil recruitment (Gabrielli et al., 2016). However, it is still unknown how Sap are sensed by neutrophils. Previous work suggests that Sap enzyme activity could create a danger signal leading to the induction of pro-inflammatory cytokines through NLRP3-inflammasome activation in host epithelial cells (Pericolini et al., 2015). Indeed, Sap were the first fungal proteins demonstrated to trigger a caspase-1 dependent NLRP3-inflammasome activation signal (Pietrella et al., 2013; Pericolini et al., 2015; Tavares et al., 2015; Roselletti et al., 2017, 2019a). In addition, it has been recently suggested that pseudohyphal rather than true hyphal cells of *C. albicans* play a critical role in VVC, possibly through the activity of multiple inflammasome inducers (Roselletti et al., 2019a). NLRP3-inflammasome activation is a two-step process described in detail elsewhere (Vanaja et al., 2015). Fungi and fungal secreted proteins, such as Sap, stimulate the release of reactive oxygen species and K^+^ efflux, which are perceived as activating signals from NLRP3 inflammasomes. Overall, these findings indicate that Sap secretion during VVC could initiate and maintain the inflammation responsible of VVC symptoms through inflammasome activation, thus perpetuating the inflammatory process through neutrophil recruitment and induction of pro-inflammatory cytokines.

### Candidalysin

Candidalysin, a fungal cytolytic peptide toxin, initially described by Moyes and coworkers, extends the list of the leading actors that play on the stage of symptomatic inflammation. Candidalysin is generated from Ece1p, a 271 amino acid polypeptide, interspersed by seven lysine-arginine repeats. The polypeptide cleavage, occurring at lysine-arginine repeats sites by Kex2p protease, results in 8 peptides of variable lengths, all secreted from the hyphae (Bader et al., 2008; Moyes et al., 2016; Richardson et al., 2018a). Of these peptides, only peptide 3 (i.e., candidalysin) has been shown to exert a lytic activity, similar to bacterial cytolysins, and to elicit innate inflammatory pathways [such as MAPK, engaged via epithelial growth factor receptor (EGFR) phosphorylation] in epithelial and endothelial cells (Ho et al., 2019). Through these and other (as yet unknown) molecular mechanisms, candidalysin directly damages epithelial membranes, triggers a danger response signaling pathway and activates epithelial immunity (Moyes et al., 2016).

Candidalysin destabilizes plasma membranes through the formation of heterogeneous and transient lesions that cause calcium influx and release of intracellular contents. In addition, the activity of this toxin has been shown to depend upon its concentration: low amounts of candidalysin trigger the release of pro-inflammatory cytokines, including granulocyte-colony stimulating factor (G-CSF), granulocyte macrophage-colony stimulating factor (GM-CSF), IL-1α, IL-1β, and IL-6. Differently, high concentrations of candidalysin can cause extensive damage in addition to pro-inflammatory response induction. Epithelial recognition of candidalysin triggers mucosal immunity predominantly through MAPK signaling, activating the p38 and ERK1/2 pathways, which in turn activate the AP-1 transcription factor c-Fos and MAPK phosphatase 1, respectively. The latter ultimately alerts the host to the transition from colonizing yeast to invasive, toxin-producing hyphae (Richardson et al., 2019).

Treatment of A-431 vaginal cells with candidalysin causes damage, MAPK/c-Fos/p-MKP1 signaling activation and cytokine secretion. Notably, candidalysin has been identified as the driver of immunopathology in the vaginal environment, since in mice challenged with fungal strains unable to express and secrete candidalysin, a significant decrease in neutrophil recruitment, damage, and pro-inflammatory cytokine expression could be observed (Richardson et al., 2018b). Candidalysin plays a crucial role in the activation of innate defenses against *C. albicans* hyphae, not only at vaginal level, but at several mucosal sites in the body.

### β-Glucan Unmasking

The last leading actor that may play a part in symptomatic inflammation is a mechanism of carbon source-mediated immune evasion developed by *C. albicans*. As elegantly demonstrated by Ballou and coworkers, the exposure to lactate, generated by host cells or bacteria of the host’s microbiota, actively triggers the masking of β-glucan, a major PAMP. Active PAMP masking in *Candida* impairs the immune system’s ability to detect the presence of the fungus. Of particular relevance, such lactate-induced β-glucan masking phenotype is specific and triggered by physiological concentrations of lactate. Carbon source-mediated immune evasion is likely to be active in the context of *Candida* vaginal colonization, since lactate levels can reach 2–4 mM in vaginal secretions (Ballou et al., 2016).

Moreover, Ene et al. (2013) had demonstrated a major impact of the carbon source upon *C. albicans* interaction with the innate immune system.

## The Concept of Neutrophil Anergy and the Role of Heparan Sulfate, pANCA Autoantibodies and CAGTA

The neutrophils recruited during VVC are not only ineffective against *C. albicans*, but they actually exacerbate the disease symptoms. The lack of anti-*C. albicans* activity by neutrophils may be due to heparan sulfate, co-colonizing bacteria, fungal factors, immune factors or a combination of them all (Gabrielli et al., 2016; Yano et al., 2017; Ardizzoni et al., 2020).

In particular, different studies suggest that specific inhibitors of neutrophil function are present in the vaginal environment during VVC. The specific nature of such inhibitors is currently a hot topic for VVC pathogenesis studies. The data obtained from a previous and very early study suggest that such inhibitor must be a protein, even though its precise identity is yet to be unraveled (Gabrielli et al., 2016).

**FIGURE 7:**
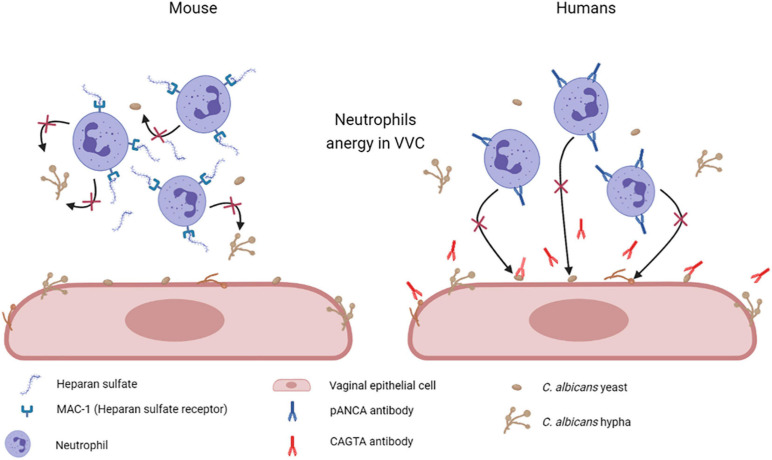
Mechanisms of neutrophil anergy during VVC. Two different mechanisms have been hypothesized to explained neutrophils anergy during VVC. It has been demonstrated in mice that heparan sulfate saturates Mac-1 receptors on neutrophils, which are necessary for fungal recognition and neutrophils mediated killing. By our recent work we have demonstrated a significant increase in anti-*C. albicans* antibodies CAGTA and autoantibodies pANCA in VVC women. pANCA in particular, are associated to neutrophils and are able to block their killing activity. Both these hypotheses are consistent with the observed neutrophils anergy in VVC (Created with BioRender.com).

Other studies indicate that heparan sulfate acts as a specific inhibitor of neutrophils function. Heparan sulfate seems to be present in the vagina of mice susceptible to chronic vaginitis and served as a competitive ligand for the neutrophilic receptor Mac-1 (also termed α_*M*_β_2_, CD11b/CD18, or complement receptor 3), which is necessary for fungal recognition and neutrophil-mediated killing. This mechanism should effectively render the PMN incapable of binding to *C. albicans* and to initiate its killing (Yano et al., 2017; Figure 7). A similar mechanism has been proposed also in women affected by VVC and RVVC, where heparan sulfate has been reported to competitively inhibit the binding of hyphal protein Pra1 (*C. albicans* pH-regulated antigen 1 protein, Pra1p) to CR3 (complement receptor 3) on neutrophils, thus impairing the efficient killing of fungal hyphae and consequently promoting *C. albicans* survival and symptomatic infection (Soloviev et al., 2011; Byrd et al., 2013; Yano et al., 2017, 2018; Bojang et al., 2021).

Based on these data, our research group has recently analyzed samples from a small number of VVC and healthy/colonized women. First, as immunological tolerance loss could be associated with differential levels of antibodies against self or commensal epitopes, we analyzed in vaginal fluids the levels of anti-*Saccharomyces cerevisiae* IgG and IgA antibodies (ASCA) that recognize mannan on the cell wall of *Saccharomycetales*. Such antibodies had been found in patients affected by diseases caused by the loss of immunological tolerance to commensal microbiota/mycobiota derived molecules (i.e., Crohn’s disease and colitis or IBD) (Xu et al., 2014; Parimelazhagan, 2016; Sommer et al., 2017). However, we found low levels only of ASCA IgA in a small group of women belonging to both groups, i.e., VVC and healthy colonized, with no significant differences.

Therefore, we analyzed the vaginal fluids for the presence of more specific anti-*C. albicans* antibodies, such as CAGTA. We found that CAGTA could be detected in VVC symptomatic women with significantly higher frequency as compared to asymptomatic *C. albicans* colonized women, demonstrating that there was an increase in *C. albicans*-specific humoral response in symptomatic patients. Then, we tried to understand why, notwithstanding the presence of *C. albicans* specific antibodies, the neutrophils remained unable to clear the fungus. Therefore, we assessed if the presence of perinuclear anti-neutrophils cytoplasmic antibodies (pANCA) could somehow impair neutrophils’ function. Interestingly, we detected significantly higher cell-associated pANCA levels in cellular fraction of vaginal fluids of symptomatic women as compared to healthy colonized women. This data is consistent with the idea that pANCA are associated to neutrophils. To support this idea, by commercially available pANCA we showed by *in vitro* experiments, that these antibodies were able to completely block the killing activity of neutrophils freshly isolated from healthy peripheral blood donors (Ardizzoni et al., 2020; Figure 7). Literature reports that pANCA directly activate neutrophils to produce ROS, thereby enhancing tissue damage (Ohlsson et al., 2014). Our data confirmed such pANCA activity in directly activating neutrophils ROS production. In addition, we demonstrated that such activity is further enhanced by concomitant challenge with *C. albicans*. Therefore, not only we demonstrated the presence of another candidate responsible for the neutrophils anergy in VVC, but we also documented a possible specific role for an auto-antibody response as the basis of the pathology.

Such novel data clearly point to a significant role of antibodies in VVC onset. Notwithstanding these first promising results, little is known about the role of other antibodies possibly occurring in vaginal fluids during VVC and their involvement in disease onset.

## Conclusion and Future Perspective: The Role of *Candida Albicans* Overgrowth and the Ineffectiveness of Pathologically Active Neutrophils (It Takes Two to Tango)

Tango is a dance from South America; it requires two partners that move in relation to each other, either in tandem or in opposition. Therefore, the title of this review, by recalling the English idiomatic expression “it takes two to tango,” suggests that in VVC a situation exists where two leading actors (i.e., the pathogen and the immune response), similarly to two tango dancers, are paired in an inextricably related and active manner. In addition, beyond its mere literal meaning, such expression implies that certain actions or activities cannot be performed alone, and they require the participation of two entities that are equally responsible for them and both are to be blamed for the eventual negative outcomes.

In other words, why is neutrophilic inflammation decoupled from fungal burden, if neutrophils are known to be efficient at killing both yeast and hyphal forms of *C. albicans*? One possibility is that an activating signal is absent from the vaginal tissue and a second possibility is that an inhibitory signal is present in this environment. According to our recent data (Ardizzoni et al., 2020), the second possibility might be the right one. Indeed, we found a significantly higher level of pANCA autoantibodies in VVC women, which presumably act as inhibitory signal causing neutrophils anergy.

In addition, from the fungal side, the dynamics that allow for its switch from a commensal to a pathogen are complex and multifactorial and they are intertwined (like in a tango) with the neutrophil’s incapacity to clear *C. albicans*, therefore exacerbating the disease symptoms. Future studies carried out on neutropenic mice will be useful to better clarify the pathogenic role of neutrophils in VVC.

**FIGURE 8:**
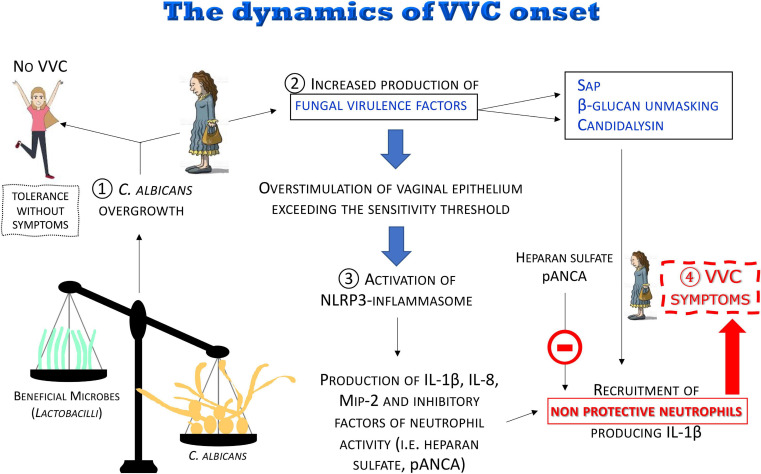
The dynamics of the VVC onset. The disruption of the vaginal microbiota may lead to *C. albicans* overgrowth with increased production of fungal virulence factors. For most women, *C. albicans* colonization is tolerated without symptoms. When the overstimulation of the epithelium exceeds the sensitivity threshold, a local intense inflammatory response is induced. Pro-inflammatory cytokines and chemokines locally produced and some fungal virulence factors (i.e., Sap, candidalysin), as well as β-glucan unmasking, induce a massive recruitment of neutrophils whose anti-fungal activity is dampened by inhibitors (i.e., heparan sulfate and pANCA) present in the vaginal environment, consequently causing the symptoms of VVC.

The most recent data and considerations summarized here are consistent with the following model (see below and Figure 8):

(1) It is believed that disruption of the microbiota balance might lead to *C. albicans* overgrowth, though the clinical outcome varies among women depending on the sensitivity of their vaginal epithelial cells. In some cases, moderate levels of *C. albicans* are tolerated in the vaginal tract: “Tolerance without symptoms”
(2) *C. albicans* overgrowth, coupled to the increased production of fungal virulence factors (i.e., Sap and candidalysin), as well as β-glucan unmasking, leads to overstimulation of vaginal epithelium, exceeding the sensitivity threshold, and to the direct induction of inflammation.
(3) The consequent uncontrolled local host inflammatory response is characterized by sustained epithelial NLRP3-inflammasome activation, pro-inflammatory cytokine production and massive infiltration of neutrophils. Neutrophils are not able to control the infection because of the presence of inhibitors in the vaginal environment such as heparan sulfate and pANCA autoantibodies.
(4) This uncontrolled local host inflammatory response ultimately causes the symptoms of VVC, i.e., itching, redness, swelling and discharge.

## Author Contributions

AA, RW, and EP wrote sections of the manuscript. All authors contributed to manuscript revision, read, and approved the submitted version.

## Conflict of Interest

The authors declare that the research was conducted in the absence of any commercial or financial relationships that could be construed as a potential conflict of interest.
